# Salivary mycobiome alterations in HIV-infected MSM: dominance of *Pseudogymnoascus* and functional shifts across disease stages

**DOI:** 10.3389/fcimb.2025.1564891

**Published:** 2025-05-09

**Authors:** Ying Guo, Lu Lin, Miao Zhang, Yixi Yu, Yan Wang, Jie Cao, Yuchen Li, Xintong Sun, Meilin Guan, Shuo Wen, Xin Wang, Zhen Fang, Wenshan Duan, Junyi Duan, Tao Huang, Wei Xia, Shan Guo, Feili Wei, Dongxiang Zheng, Xiaojie Huang

**Affiliations:** ^1^ Beijing Institute of Hepatology, Beijing Youan Hospital, Capital Medical University, Beijing, China; ^2^ Department of Stomatology, Beijing Youan Hospital, Capital Medical University, Beijing, China; ^3^ Clinical and Research Center for Infectious Diseases, Beijing Youan Hospital, Capital Medical University, Beijing, China

**Keywords:** saliva, mycobiome, PLWHA, MSM, metagenomic analyses, KEGG function

## Abstract

**Background:**

Oral health is increasingly recognized as a crucial determinant of overall health in people living with HIV/AIDS (PLWHA). Specifically, the oral mycobiome may play a pivotal role in HIV-associated oral complications. However, the fungal species involved and their potential as biomarkers for HIV-related oral conditions remain poorly understood. This study investigates salivary fungal profiles in PLWHA who have sex with men (MSM), focusing on diversity, functional shifts, and correlations with disease progression.

**Methods:**

A cross-sectional study included 25 MSM participants divided into five groups: HIV-negative controls (n = 5) and four HIV-positive groups stratified by CD4 count: Stage 0 (HIV RNA-positive/antibody-negative; n = 5), Stage 1 (CD4 ≥500 cells/μL; n = 5), Stage 2 (CD4 200–499 cells/μL; n = 5), and Stage 3 (CD4 <200 cells/μL or opportunistic infections; n = 5). Saliva samples were collected and analyzed using metagenomic sequencing (Illumina NovaSeq platform). Bioinformatic analyses included genome assembly (MEGAHIT), gene clustering (CD-HIT), gene abundance calculation (SOAPaligner), species annotation (BLASTP), and KEGG pathway annotation (KOBAS 2.0). Statistical analyses (Kruskal-Wallis tests, Spearman’s correlation) assessed associations between fungal profiles, CD4 count, and viral loads.

**Results:**

A total of 51 fungal genera were identified, with *Pseudogymnoascus* being the most abundant. Functional analysis revealed 113 shared KEGG pathways, of which 69 were unique to Stage 3, primarily related to metabolic and disease-related processes. Notably, *Auricularia* exhibited a positive correlation with CD4 count (*P* ≤ 0.01), while *Mucor* showed a negative correlation (*P* = 0.0299).

**Conclusions:**

Salivary mycobiome composition and function shift significantly across HIV stages, reflecting immune decline. *Pseudogymnoascus* dominance challenges conventional views of oral fungal ecology in immunocompromised hosts. These findings highlight the mycobiome’s diagnostic potential for monitoring HIV-related oral health. Longitudinal studies are needed to validate clinical relevance.

## 1 Introduction

Oral health is critically important for people living with HIV/AIDS (PLWHA), as they are particularly vulnerable to oral diseases, especially fungal infections such as candidiasis (Mushi et al., 2017; Spalanzani et al., 2018; Bhattacharya et al., 2020; Fidel et al., 2021; Lu, 2021; Erfaninejad et al., 2022; Ngozi and Ifeyinwa, 2022). Among these, *Candida* species *-* particularly *Candida albicans*—typically exist as commensals in the oral cavity but can become pathogenic in immunocompromised individuals, leading to complications like oral candidiasis (Metsch et al., 2015; Annavajhala et al., 2020; Vila et al., 2020; d’Enfert et al., 2021; Talapko et al., 2021; Jaqua et al., 2022; Lopes and Lionakis, 2022). The oral microbiome is a complex ecosystem comprising fungi, bacteria, viruses, and archaea that maintain a delicate balance. In HIV-infected individuals, this balance often becomes disrupted, leading to opportunistic infections that further compromise the immune system. Despite this, research into oral fungi in HIV patients remains limited, with a disproportionate focus on bacterial studies (Hager and Ghannoum, 2018; Sodré et al., 2020; Fidel et al., 2021; Erfaninejad et al., 2022).

While some studies have explored the fungal communities within the oral microbiota of HIV-infected individuals, most focus on the diversity analyses, primarily describing species richness and compositional differences (Mukherjee et al., 2014; Annavajhala et al., 2020; Chang et al., 2021). Current findings suggest that the oral fungal composition in HIV-infected patients may differ significantly from that of healthy controls, exhibiting both increased diversity and higher fungal abundance. Candida species commonly dominate the oral fungal profiles of HIV-infected individuals, a trend observed regardless of their antiretroviral therapy (ART) status (Mukherjee et al., 2014; Annavajhala et al., 2020). Additionally, smoking may influence the diversity of oral fungal flora in this population (Mukherjee et al., 2018). Some evidence also suggests that patients who had previously been diagnosed with AIDS exhibit reduced fungal α-diversity in both saliva and dental plaque. After ART initiation, a decline in fungal diversity occurs, closely associated with immune activation (Annavajhala et al., 2020). These changes are likely driven by the immunosuppressive effects of HIV, which could promote fungal colonization and overgrowth (Chang et al., 2021).

Metagenomic analysis provides a robust tool for characterizing microbial communities by sequencing genetic material directly from clinical samples. Shotgun metagenomics enables comprehensive profiling of the oral mycobiome, resolving taxonomic composition and functional potential, including metabolic pathways and ecological roles (Pérez-Cobas et al., 2020; Xie et al., 2023). This approach facilitates detection of fungi, including those that cannot be cultured. However, metagenomic approaches remain underutilized in the study of salivary fungi among HIV-infected individuals, limiting our understanding of fungal diversity and pathogenic potential in the oral environment. This knowledge gap hampers our comprehension of how fungal communities evolve across different stages of HIV infection and their implications for oral health.

To address these gaps, comprehensive metagenomic studies are essential. Such research could deepen our understanding of the role of oral fungi in the health and disease of PLWHA, providing insights into how these fungal populations shift over time and contribute to oral health. Expanding our knowledge of these microbial communities is crucial for a complete understanding of the oral ecosystem in HIV patients. Therefore, this study aims to investigate stage-specific alterations in the salivary mycobiome across HIV disease progression, with a focus on functional shifts and biomarker potential. We hypothesize that (1) fungal community structure and metabolic pathways differ significantly among HIV stages stratified by CD4 count, and (2) key fungal taxa correlate with immunological decline and serve as stage-specific biomarkers.

## 2 Materials and methods

### 2.1 Study design

This cross-sectional study involved men who have sex with men (MSM) from Beijing, China. Participants were divided into five groups based on HIV status and CD4 counts according to CDC definitions (Centers for Disease Control and Prevention (CDC), 2014), as follows:

Control (HIV_neg): HIV-negative MSM.
Stage 0 (HIV_0): HIV RNA-positive but antibody-negative/indeterminate.
Stage 1 (HIV_1): CD4 count ≥500 cells/μL.
Stage 2 (HIV_2): CD4 count 200–499 cells/μL.
Stage 3 (HIV_3): CD4 count <200 cells/μL or presence of opportunistic infections.

### 2.2 Ethical approval and participant consent

The study was approved by the Institutional Review Board of Beijing Youan Hospital (Approval No. JYKL-[2017]-30), and written informed consent was obtained from all participants before enrollment.

### 2.3 Inclusion and exclusion criteria

The inclusion criteria were as follows: (1) Male patients aged ≥18 years; (2) Confirmed HIV infection through serological testing; (3) No current antiretroviral therapy (ART) regimen; (4) No use of immunomodulatory drugs; (5) No antibiotic use within the last three months; (6) No significant oral health issues, except for non-cavitated caries, non-purulent periodontal disease, or oral candidiasis; and (7) Presence of at least 20 teeth.

Exclusion criteria were as follows:(1) History of systemic fungal infections; (2) Recent hospitalization (within the last three months); (3) Diagnosis of autoimmune diseases or malignancies; (4) Recent corticosteroid or immunosuppressive therapy within the past three months; (5) Known hypersensitivity to the sample collection materials; and (6) Incomplete clinical data or lack of adherence to study protocols.

### 2.4 Samples collection

Participants were instructed to refrain from consuming food or beverages and using oral hygiene products for at least two hours before sample collection to minimize external contamination. Saliva samples were collected in the morning before any oral activity. Participants waited for 5-20 minutes to allow for baseline saliva production. Unstimulated whole saliva was collected by passive drooling into a sterile tube until at least 5 mL was obtained. Immediately following collection, samples were placed on ice. To prevent repeated freeze-thaw cycles, all samples were processed within two hours and stored at -80°C until DNA extraction.

### 2.5 DNA extraction and sequencing

Genomic DNA was extracted from 500 μL of each saliva sample using the E.Z.N.A.^®^ Soil DNA Kit (Omega Bio-tek, U.S.), following the manufacturer’s instructions. DNA concentration and purity were assessed using a TBS-380 fluorometer and NanoDrop2000 spectrophotometer, respectively. DNA was fragmented to approximately 300 bp using the Covaris M220 (Gene Company, China). A paired-end library was constructed using the NEXTFLEX^®^ Rapid DNA-Seq kit (Bioo Scientific, USA). Sequencing was conducted on the Illumina NovaSeq platform (Illumina, USA) at Majorbio Bio-Pharm Technology Co., Ltd. (Shanghai, China). The relevant data are deposited in the NCBI Short Read Archive database (SRP327008).

### 2.6 Bioinformatics analysis

Quality trimming of reads was performed using Fastp v0.20.0 (https://github.com/OpenGene/fastp). Contaminant reads were removed using alignment with BWA (http://bio-bwa.sourceforge.net, version 0.7.9a). Genome assembly was conducted with MEGAHIT (https://github.com/voutcn/megahit, version 1.1.2), which efficiently handles large-scale metagenomic data. A non-redundant gene set was generated using CD-HIT (http://www.bioinformatics.org/cd-hit/, version 4.6.1). Gene abundance was calculated with SOAPaligner (http://soap.genomics.org.cn/, version 2.21), species annotation was performed using BLASTP (BLAST Version 2.2.28+, http://blast.ncbi.nlm.nih.gov/Blast.cgi), and KEGG functional annotation was performed using KOBAS 2.0 (KEGG Orthology Based Annotation System, http://kobas.cbi.pku.edu.cn/home.do).

### 2.7 Statistical analysis

Quantitative data following a normal distribution are presented as means with standard deviations (mean ± SD), and comparisons between two groups were conducted using the t-test. If the data did not follow a normal distribution, they are reported as medians with interquartile ranges (median [IQR]), and the Wilcoxon rank-sum test was used for comparisons between groups. Categorical data are presented as frequencies with percentages (n [%]), and comparisons between groups were performed using the chi-square test.

Fungal gene sets were constructed based on taxonomic annotation results of metagenomic data, and salivary fungal abundance was normalized using reads per kilobase per million mapped reads (RPKM). β-diversity analysis was performed by calculating genus-level community dissimilarities with the Pearson distance metric. The Kruskal-Wallis rank-sum test (two-tailed) was applied to assess the significance of structural differences in microbial communities among the five groups. Intergroup variations in fungal genera and KEGG functional profiles were analyzed using the Kruskal-Wallis test with false discovery rate (FDR) correction, followed by Games-Howell *post-hoc* testing (significance threshold α = 0.05). Spearman’s correlation coefficients were employed to evaluate associations between the top 40 most abundant fungal genera/functions and CD4 counts or blood viral load (BVL) in the HIV-positive group.

## 3 Results

### 3.1 Participant demographics

This study adhered strictly to the inclusion and exclusion criteria, resulting in the selection of 25 MSM participants from Beijing, including 20 PLWHA. Participants were stratified into five groups based on their CD4 counts, viral load, and western blot confirmation tests, as outlined in **Table 1**. Importantly, there were no significant differences in age, smoking behavior, periodontal health, or mucosal condition between the five groups (*P* > 0.05). Two participants in Stage 3 presented with mild pseudomembranous candidiasis, although both were asymptomatic. Informed consent was obtained from all participants prior to the collection of salivary samples.

**Table 1:** Demographic and clinical characteristics of MSM participants by HIV stage (n=5 per group).

Group	HIV_0 (Stage 0)	HIV_1 (Stage 1)	HIV_2 (Stage 2)	HIV_3 (Stage 2)	HIV_neg (Control)	Chi-square/Z value	*P* value
Age, years	31 (25.50, 43.00)	34 (24.50, 39.50)	42 (30.50, 46.00)	29 (22.50, 39.00)	29 (24.50, 39.00)	3.45	0.49
Smoking status, n (%)							
Non-smoker	5(100.00)	4 (80.00)	2(40.00)	3 (60.00)	5(100.00)	9.09	0.06
Smoker	0 (0.00)	1 (20.00)	3 (60.00)	2(40.00)	0 (0.00)		
Periodontal status, n (%)							
Periodontal health	1 (20.00)	2(40.00)	1 (20.00)	1 (20.00)	1 (20.00)	0.81	0.94
Periodontitis	4 (80.00)	3(60.00)	4 (80.00)	4 (80.00)	4 (80.00)		
Mucosal status, n (%)							
Mucosal health	5(100.00)	5(100.00)	5(100.00)	3 (60.00)	5(100.00)	7.21	0.13
Oral candidiasis	0 (0.00)	0 (0.00)	0 (0.00)	2(40.00)	0 (0.00)		
CD4 counts cells/μL	354.33 (327.52, 470.52 )	675.08 (604.52, 761.58)	243.00 (219.77, 374.73)	186.85 (63.44, 234.04)	NA	15.75	<0.01
Blood Viral Load log_10,_ copies/mL	4.28 (3.93, 4.94)	3.83 (3.47, 4.67)	5.17 (4.60, 5.47)	4.34 (3.97, 5.40)	0 (0.00, 0.00)	15.01	<0.01

MSM, men who have sex with men; NA: Not available.

### 3.2 Salivary fungal profile in MSM living with HIV/AIDS

**Figure 1:**
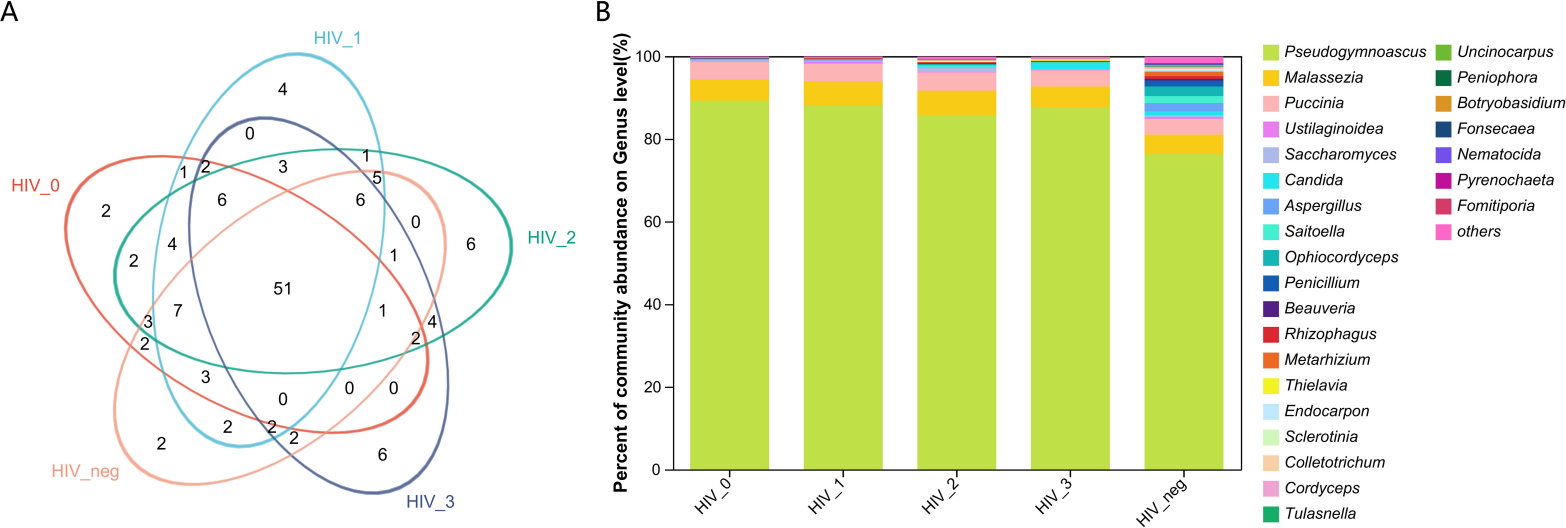
Composition and abundance of salivary fungal microbiota at the genus level. **(A)** The Venn diagram displays the overlap of fungal genera across five sample groups. The numbers within the overlapping areas of the diagram represent the shared genera, while non-overlapping areas indicate unique genera. **(B)** The community barplot illustrates the composition and relative abundance of the top 26 genera across the sample groups, with lower-abundance genera grouped as “Others”. The x-axis represents the five study groups, while the y-axis indicates the proportion of each genus. Different colours represent different genera, and the length of each bar corresponds to the proportion of the respective genus.

The salivary fungal microenvironment in PLWHA was analyzed from the perspective of genus composition. **Figures 1A, B** provide an overview of the fungal community structure. The Venn diagram displays the distribution of genera across stages 0 to 3, with 86, 97, 102, and 86 genera identified in each stage, respectively. The negative control group contained 87 genera. Notably, 51 genera were shared among all five groups, with each group presenting a similar number of unique genera. The 0 and negative control groups each exhibited 2 unique genera, while the number of unique genera in Stages 1 to 3 increased gradually from 4 to 6, with Stages 2 and 3 having an identical number. The bar chart further illustrates the composition of fungal genera across the groups. *Pseudogymnoascus* was the most abundant genus among the 26 identified, followed by *Malassezia*, *Puccinia*, *Ustilaginoidea*, *Saccharomyces*, *Candida*, *Aspergillus*, *Saitoella*, *Ophiocordyceps*, and *Penicillium*. A closer examination reveals that the fungal composition in the four HIV-positive groups is generally consistent, but notably distinct from that of the negative control group. All detected genera and their relative abundances are detailed in **Supplementary Table S1**.

### 3.3 Salivary fungal KEGG functional potential in MSM living with HIV/AIDS

**Figure 2:**
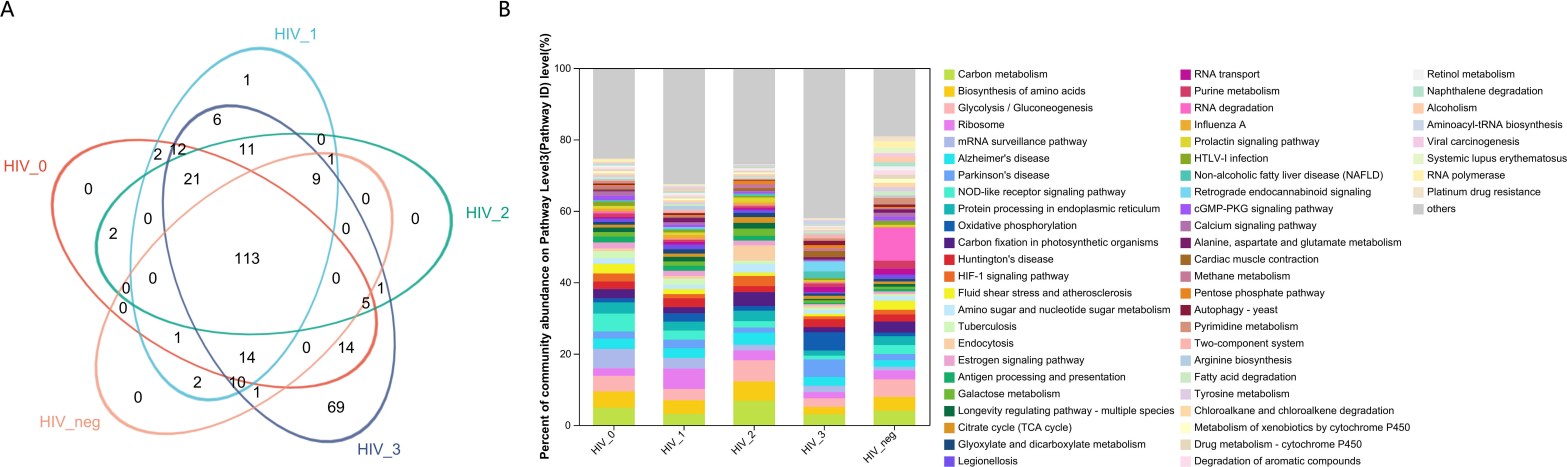
Functional composition and abundance of salivary fungal microbiota at KEGG Pathway Level 3. **(A)** The Venn diagram displays the overlap of fungal functions across five sample groups.The numbers within the overlapping areas of the diagram represent the shared functions, while non-overlapping areas indicate unique functions. **(B)** The community bar plot illustrates the composition and relative abundance of the top 56 KEGG functions across samples, with lower-abundance fungal genera categorized as “Others”. The x-axis represents the five study groups, while the y-axis indicates the proportion of each function within each group. Different colours denote different functions, with the length of each bar representing the relative proportion of that function.

**Figure 3:**
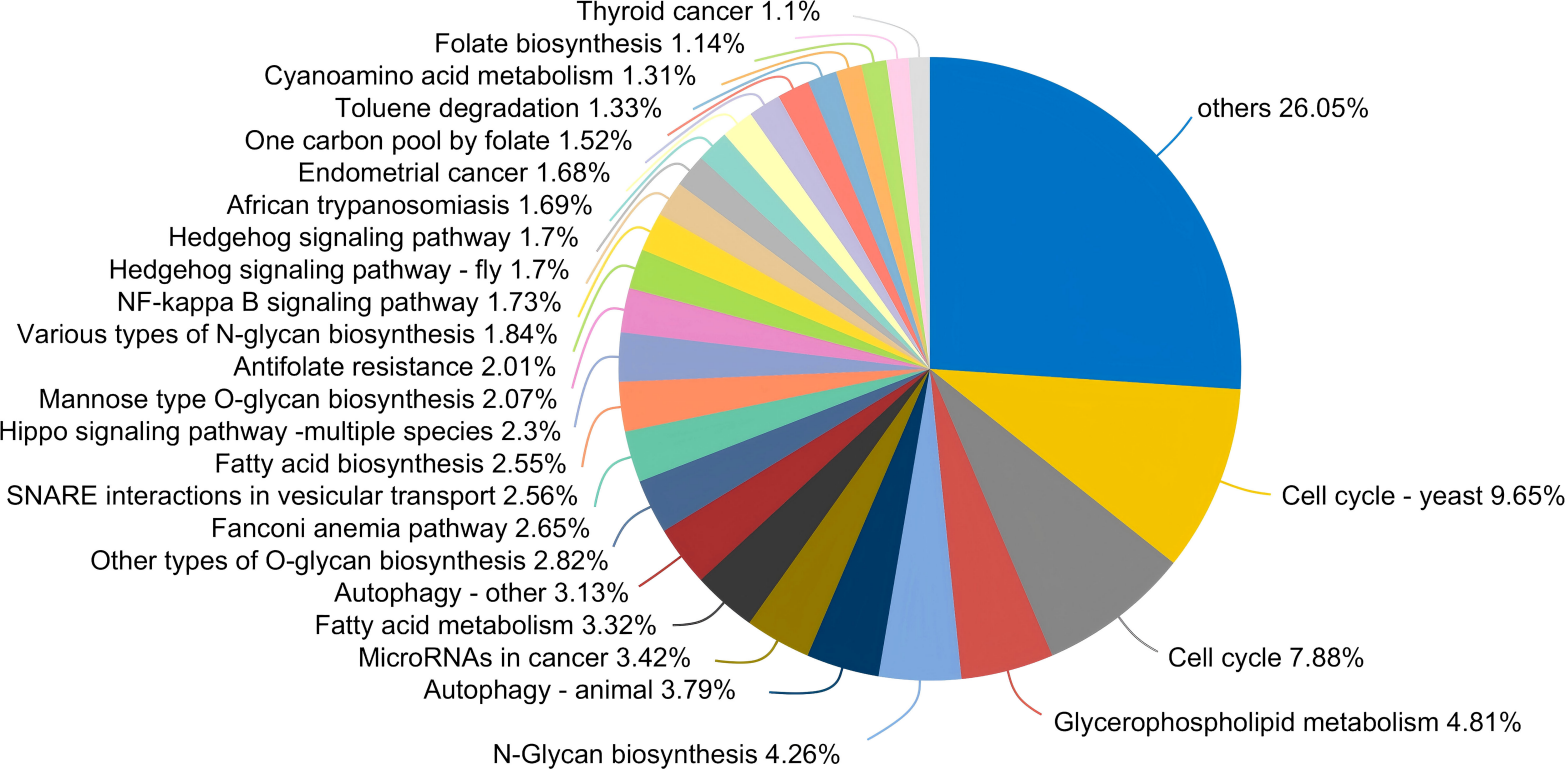
Pieplot illustrating KEGG functions unique to the salivary fungal microbiome in the Stage 3 group at Pathway Level 3. Different colours represent distinct functions, and the size of each segment corresponds to the percentage of that function relative to the total number of functions.

From a functional perspective, the salivary fungal microenvironment in PLWHA displays distinct characteristics based on fungal KEGG gene pathways. As depicted in the Venn diagram (**Figure 2A**), there are 113 shared fungal functions across all groups, with the Stage 3 group exhibiting the highest number of unique functions, totaling 69 (**Figure 3**, **Supplementary Table S2**). These unique functions are classified into the following Pathway Level 1 categories: 4 in Genetic Information Processing, 6 in Cellular Processes, 8 in Environmental Information Processing, 12 in Organismal Systems, 14 in Human Diseases, and 25 in Metabolism.

In contrast, the control, Stage 0, and Stage 2 groups displayed no unique functions, while the Stage 1 group had only one unique function. This indicates a substantially different functional profile compared to the composition of fungal genera observed across the various stages. The bar chart (**Figure 2B**) highlights predominant functions at Pathway Level 3 for the MSM subjects, including Carbon Metabolism, Biosynthesis of Amino Acids, Glycolysis/Gluconeogenesis, Ribosome, mRNA Surveillance Pathway, Alzheimer’s Disease, Parkinson’s Disease, and NOD-like Receptor Signaling Pathway. Detailed functional relative abundance distributions are provided in **Supplementary Table S3**. This functional divergence underscores the complexity of the oral mycobiome across different stages of HIV infection and its potential implications for disease progression and oral health.

### 3.4 Differences in salivary fungal genera and KEGG function across disease stages in MSM living with HIV/AIDS

**Figure 4:**
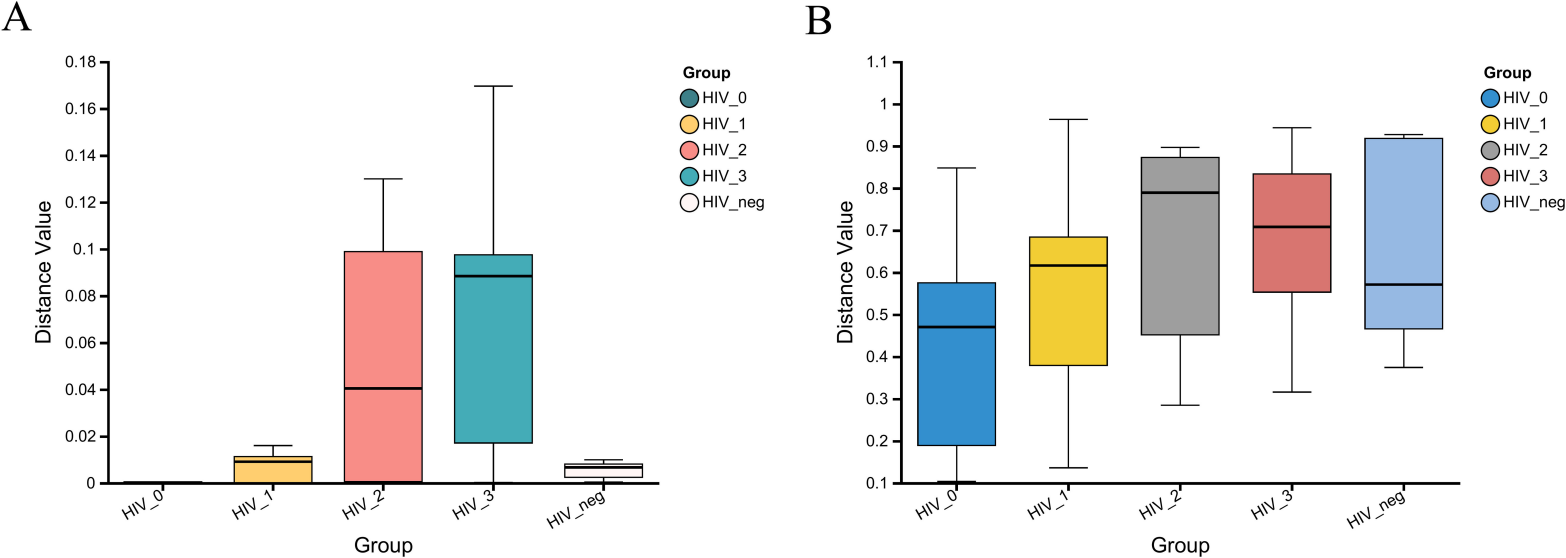
β-diversity analysis of salivary fungal microbiota across five groups, evaluated using the Kruskal-Wallis rank sum test. **(A)** Variation at the genus level (*P* = 6.148e-05). **(B)** Variation at Pathway level 3 functional analysis (*P* = 0.2152). The x-axis represents the five groups, with the box plots indicating the distribution within each group. The y-axis shows the β-diversity distance values within the groups.

The Kruskal-Wallis rank sum test was employed to assess the within-group dispersion of salivary fungal samples across five groups by analyzing β-diversity differences. The boxplot in **Figure 4** illustrates the degree of dispersion within each group. At the fungal genus level, dispersion was notably higher in the Stage 2 and Stage 3 groups, while it was lower in the Stage 0 and control groups. However, at the KEGG functional level, dispersion appeared relatively consistent across all groups. The β-diversity analysis revealed significant differences at the fungal genus level (*P* = 6.148e-05), but no significant differences were observed at the Pathway Level 3 functional level (*P* = 0.2152). These results suggest substantial differences in the composition of salivary fungal communities among the five groups, whereas the KEGG functions at Pathway Level 3 do not show significant variation.

**Figure 5:**
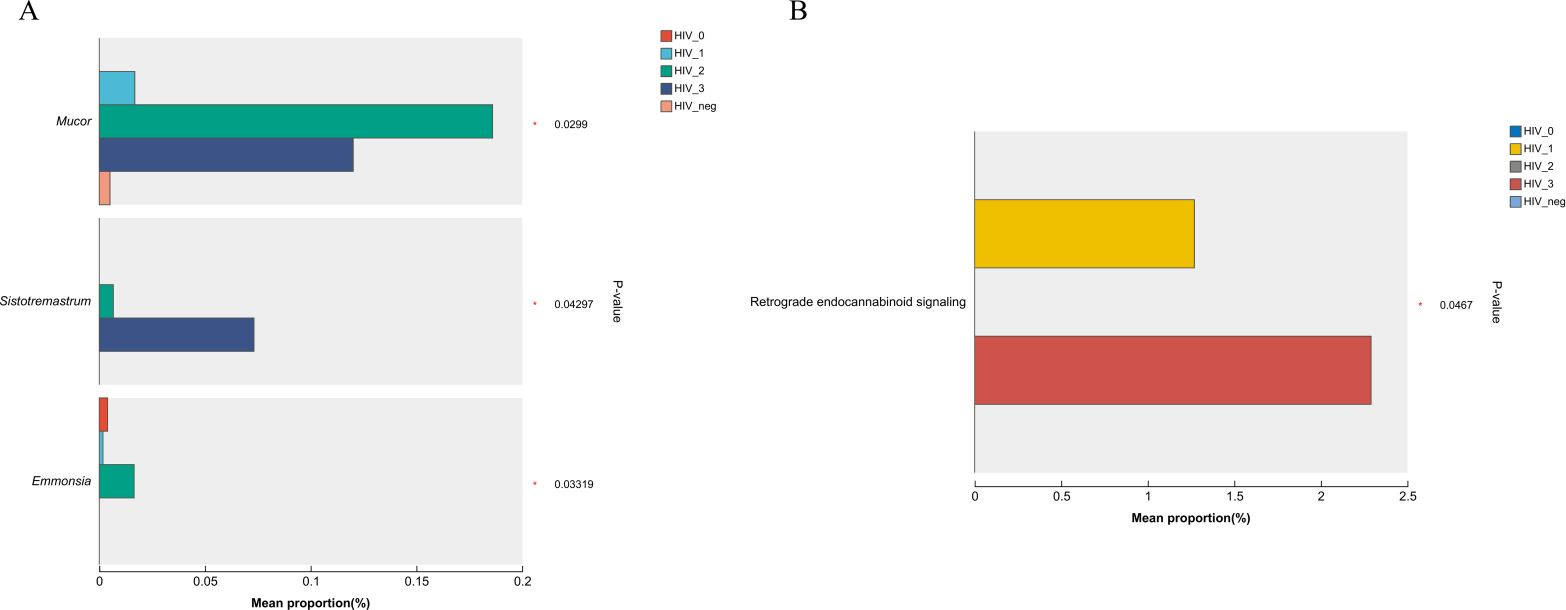
Bar plots showing differences in salivary fungal microbiota among five MSM groups, analyzed using the Kruskal–Wallis rank sum test. (A) Differential fungal genera; (B) Differential KEGG level 3 functional pathways. The x-axis shows the percentage abundance of specific genera or KEGG functions within the samples, while the y-axis lists the genera/functions exhibiting significant differences. Different colours represent the various groups. Significance levels are indicated as: 0.01 < *P* ≤ 0.05 (*).

In light of the observed differences in microbial communities among the five groups, the Kruskal-Wallis rank sum test was applied to compare the relative abundances of fungal genera and KEGG functions across these groups. As shown in the bar chart (**Figure 5A**), the fungal genera *Mucor* (*P* = 0.0299), *Sistotremastrum* (*P* = 0.04297), and *Emmonsia* (*P* = 0.03319) were significantly more abundant in the positive group. Furthermore, the bar chart in **Figure 5B** demonstrates significant differences in the Pathway Level 3 function of Retrograde endocannabinoid signaling (*P* = 0.0467) between groups, with higher abundance of this pathway also observed in the positive group.

### 3.5 Correlation between salivary fungal genera/KEGG function and immune markers in MSM living with HIV/AIDS

**Figure 6:**
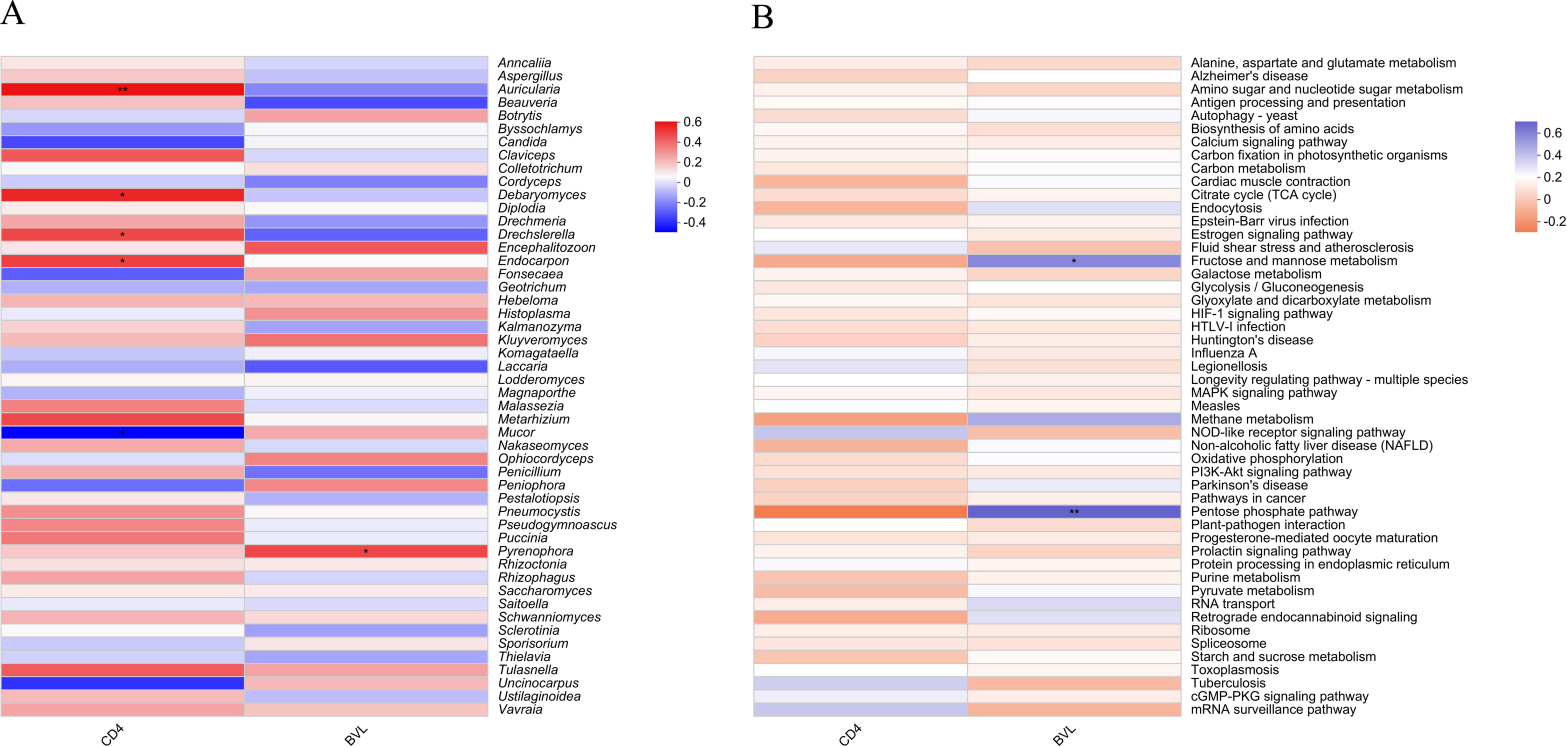
Heatmap showing the correlation between the top 50 salivary fungal microbiota by taxonomic abundance and CD4 count or BVL in PLWHA. **(A)** Fungal genera; **(B)** Pathway level 3 functions. The X-axis represents immune markers of PLWHA, while the Y-axis shows fungal genera/functions in the salivary microbiota. The color in the upper part of each box indicates a positive correlation, while the lower part indicates a negative correlation. Significant correlations are denoted by *: 0.01 < *P* ≤ 0.05 *, 0.001 < *P* ≤ 0.01**.

The correlation heatmap demonstrates the relationships between CD4 count, BVL, and the top 50 most abundant fungal genera or functions identified in salivary samples from HIV-positive individuals. In **Figure 6A**, four fungal genera exhibit significant positive correlations with CD4 count: *Auricularia* (*P* ≤ 0.01), *Debaryomyces* (*P* ≤ 0.05), *Drechslerella* (*P* ≤ 0.05), and *Endocarpon* (*P* ≤ 0.05). Conversely, *Mucor* (*P* ≤ 0.05) is the only genus significantly negatively correlated with CD4 count. In relation to BVL, only *Pyrenophora* (*P* ≤ 0.05) shows a significant positive correlation. At the pathway level 3 (**Figure 6B**), Fructose and mannose metabolism (*P* ≤ 0.05) and Pentose phosphate pathway (*P* ≤ 0.01) are the two functions significantly positively correlated with BVL.

## 4 Discussion

Fungi, while often regarded as minor components of the oral microbiome, play a significant role in maintaining oral health, particularly in PLWHA. Their pathogenic potential typically becomes evident only under conditions that favor opportunistic infections, which contributes to their limited study compared to bacteria. In PLWHA, fungi are crucial for balancing oral health and disease, with the occurrence of candidiasis often serving as a clinical marker for the progression of HIV to AIDS. Previous research on oral fungi has primarily focused on individual pathogenic species, often drawing from clinical case reports (Bandara and Samaranayake, 2019; Mutalik et al., 2021; Roomaney et al., 2021; Santosh et al., 2021; Devi et al., 2024). However, recent advances in DNA sequencing technologies have shifted the focus towards a more holistic understanding of the oral mycobiome. Despite this progress, challenges such as low sensitivity in detecting certain fungal taxa remain (Baldrian and Lepinay, 2021; Cannon, 2022). To address this, we employed high-depth metagenomic RNA sequencing, which enables precise taxonomic identification down to the species level, particularly for the most abundant species with complete genomes available in databases (Aragona et al., 2022). This approach facilitates the identification of viable but non-culturable (VBNC) fungi, even though it cannot confirm their cultivability (Wu et al., 2019). Nevertheless, it provides a valuable tool for clinical research, offering deeper insights into the composition and functional potential of the oral mycobiome.

In this study, we used metagenomic techniques to examine the composition of salivary fungi in untreated PLWHA across various stages of HIV infection. Notably, *Pseudogymnoascus* emerged as the most abundant genus in all participant groups, contrasting with the expected prominence of *Candida* in the oral mycobiome of immunocompromised populations. This finding differs from observations in similar studies, such as those on cancer patients, where *Candida* and *Malassezia* are predominant components of the salivary fungal community (Hong et al., 2020). In our study, *Candida* ranked only sixth in abundance, challenging traditional views of the oral mycobiome in immunocompromised individuals.

The salivary fungal profile of the HIV-negative MSM control group included 87 genera, aligning with data from healthy populations (Ghannoum et al., 2010), though the dominant genera showed considerable differences. *Pseudogymnoascus* was the most abundant genus across all MSM groups, including both HIV-positive and negative participants, which contrasts with previous reports from healthy individuals (Ghannoum et al., 2010). This discrepancy raises the possibility that unique behavioral factors associated with the MSM population, including sexual behavior patterns, may contribute to shifts in the oral fungal community structure. Further comparative analysis is warranted to clarify these potential influences. Interestingly, the predominance of *Pseudogymnoascus*, primarily recognized as a bat pathogen (Villanueva et al., 2021; Isidoro-Ayza and Klein, 2024), in HIV-positive MSM individuals suggests that shifts in oral fungal ecology may be linked to behavioral factors unique to the MSM population or methodological variations in fungal detection. *Pseudogymnoascus destructans*, an important psychrophilic bat pathogen, has been shown to survive at 37°C for up to 15 days under artificial cultivation conditions suggesting that it may have the potential for direct transmission between hosts under certain conditions (Campbell et al., 2020), indicating potential ecological plasticity and the capacity for direct transmission between hosts under certain environmental conditions. Beyond its recognized pathogenic role in animals, emerging evidence indicates that *Pseudogymnoascus* may have broader implications for human health. For example, *Pseudogymnoascus destructans* has been associated with a lower pathological stage in head and neck squamous cell carcinoma (Chakladar et al., 2022), suggesting a potential protective or modulatory role in carcinogenesis. Additionally, sesquiterpenoids derived from *Pseudogymnoascus* sp. have exhibited cytotoxic effects against various human cancer cell lines, including breast (MDA-MB-231), colorectal (HCT116), and hepatoma (HepG2) cells (Shi et al., 2021), highlighting its potential dual role as both a commensal component of the oral mycobiome and a factor influencing cancer development.

Although the abundance of *Pseudogymnoascus* was slightly higher in HIV-positive participants compared to controls, this difference was not statistically significant, suggesting that immune alterations in PLWHA may have a limited impact on *Pseudogymnoascus* colonization. However, this finding does not rule out the possibility of more subtle immune-mediated effects that may influence fungal ecology over time. Furthermore, the potential for bacterial-fungal symbiosis in the oral mycobiome warrants further exploration. The potential for bacterial-fungal symbiosis in the oral mycobiome warrants further exploration (Shirtliff et al., 2009). Cross-kingdom interactions between *Candida albicans* and *Helicobacter pylori* have been documented, where *C. albicans* serves as a protective reservoir and transmission vehicle for *H. pylori.* Internalization into *C. albicans* may enhance *H. pylori* survival in hostile environments, increasing its transmission potential (Chen et al., 2021). Similar mechanisms may apply to *Pseudogymnoascus*, potentially explaining its dominance in the HIV-infected oral environment. The presence of other co-cultured oral microorganisms, such as *Candida dubliniensis*, could further facilitate the persistence and spread of *H. pylori*, playing a necessary role in its infection cycle and transmission (Scholz et al., 2025). These interactions underline the complexity of microbial relationships within the oral cavity and suggest that *Pseudogymnoascus* may engage in analogous symbiotic or protective roles. Co-culture systems and single-cell sequencing techniques could provide deeper insights into these cross-kingdom interactions, helping to clarify the ecological dynamics within the oral mycobiome.

The core fungal microbiome remained stable across all stages of HIV infection, characterized by substantial overlap in genera, which indicates that overall diversity was relatively consistent. However, subtle shifts in community composition may reflect the host’s immune status changes. Variations in genus abundance could represent adaptive responses of the salivary fungal community to the dynamic immune environment associated with HIV progression. Our functional analysis revealed significant reorganization within the salivary fungal microbiome, particularly in advanced stages of HIV infection. Participants in Stage 3 exhibited 69 unique functions, predominantly related to metabolic pathways and disease processes, suggesting an association between severe metabolic disorders and late-stage HIV. These findings have potential implications for systemic diseases, including Alzheimer’s and Parkinson’s. The functional changes observed may reflect adaptive or compensatory responses of the fungal community to altered host immunity and modifications in the oral microenvironment. While our beta diversity analysis indicated significant compositional differences in fungal genera across groups, functional consistency was largely maintained. This suggests that even marked changes in fungal genera do not necessarily translate into broad functional reorganization. However, the emergence of unique functions in Stage 3 may imply that prolonged immunosuppression could drive fungi to contribute to secondary infections or malignancies, warranting further investigation.

The substantial variations in fungal genera and metabolic pathways across groups highlight the dynamic nature of the salivary mycobiome in PLWHA. The increased abundance of *Mucor, Sistotremastrum, and Emmonsia* in the HIV-positive group points to their potential association with immunocompromised conditions characteristic of PLWHA. *Mucor* species are known opportunistic pathogens (Chen et al., 2022; Muthu et al., 2023) linked to *mucormycosis*, while *Emmonsia* has been associated with systemic infections in immunocompromised individuals (Govender and Grayson, 2019; Madani and Grayson, 2022). Our findings support the notion that these genera could serve as biomarkers for HIV-related oral health complications. Additionally, the significant differences observed in KEGG Pathway Level 3 functions, particularly concerning retrograde endocannabinoid signaling, indicate potential metabolic alterations associated with HIV infection. This pathway is integral to neuromodulation and inflammation regulation (Chu et al., 2023), both of which are pivotal in the context of HIV-associated neurocognitive disorders. The increased presence of this pathway in HIV-positive participants may reflect a compensatory mechanism or dysregulation driven by persistent immune challenges.

Furthermore, correlations between specific fungal genera and CD4 counts provide insights into their potential role in oral health among immunocompromised individuals. The positive correlation of genera like *Auricularia* with CD4 counts suggests that certain low-abundance fungi might contribute to maintaining a balanced oral mycobiome. Conversely, the negative correlation of *Mucor* with CD4 counts highlights its potential role in opportunistic infections during advanced AIDS stages. Interestingly, similar findings have been reported in tongue cancer research, where elevated *Mucor* levels were linked to weakened immunity, suggesting that immune dysfunction may create a favorable niche for *Mucor* colonization (Mukherjee et al., 2017). Notably, bacterial dysbiosis in the oral cavity has also been associated with increased susceptibility to invasive mucormycosis (Vallianou et al., 2021). The disruption of microbial balance may weaken mucosal defenses, facilitating fungal overgrowth and pathogenicity. These findings underscore the complex interplay between bacterial and fungal communities in shaping the oral microenvironment, particularly in immunocompromised individuals. The upregulation of fructose and mannose metabolism (Zhang et al., 2021), alongside the pentose phosphate pathway (Shytaj et al., 2021; Crater et al., 2022), indicates heightened metabolic demands driven by viral replication, likely fostering inflammation and immune activation. These findings emphasize the significance of metabolic pathways in host-fungal interactions in HIV-positive individuals, highlighting potential therapeutic targets aimed at modulating these pathways to enhance patient outcomes.

This study has several limitations that should be noted. First, the sample size of 25 MSM participants restricts the generalizability of our findings. Additionally, the inclusion of only male participants limits the applicability of our results to a broader population. Future studies should aim to include diverse demographic groups, particularly female participants, to improve the comprehensiveness of our findings. Second, the cross-sectional design of this study precludes causal inferences regarding the relationship between HIV progression and changes in the salivary mycobiome. Longitudinal studies will be necessary to establish temporal associations and better understand the dynamics of fungal community shifts over time. Third, while metagenomic sequencing provided valuable insights, detection sensitivity issues may have led to an underrepresentation of low-abundance fungi taxa. Moreover, we did not systematically collect patient age data, which could serve as an additional factor influencing oral mycobiome composition. Finally, although we did not impose restrictions on oral hygiene habits, periodontal health was assessed as an objective indicator of oral conditions. While this provides useful insights, it does not fully account for individual variations in oral hygiene practices. We have acknowledged this as a limitation and suggest that future studies incorporate more detailed assessments of oral hygiene behaviors alongside other potential confounders such as dietary habits and concurrent oral infections. Future research should account for these variables and employ larger, longitudinal cohorts to enhance the clinical relevance of the observed microbial and functional changes.

## 5 Conclusion

In conclusion, our study highlights the complex role of the oral mycobiome in untreated PLWHA, with an unexpected predominance of *Pseudogymnoascus* and significant functional shifts observed in advanced HIV stages. The stability of core genera, coupled with metabolic changes, suggests adaptive responses to immune status alterations. The increased presence of *Mucor* and *Emmonsia*, along with associated metabolic shifts, indicates their potential as biomarkers for HIV-related oral health issues. However, the limitations of our study, particularly the small sample size and cross-sectional nature, warrant further investigation to confirm these findings and explore their broader biological significance.

## Data Availability

The datasets presented in this study can be found in online repositories. The names of the repository/repositories and accession number(s) can be found below: https://www.ncbi.nlm.nih.gov/, SRP327008.

## References

[B1] Annavajhala M. K. Khan S. D. Sullivan S. B. Shah J. Pass L. Kister K.. (2020). Oral and gut microbial diversity and immune regulation in patients with HIV on antiretroviral therapy. mSphere 5, e00798–e00719. doi: 10.1128/mSphere.00798-19 PMC7002309 32024712

[B2] Aragona M. Haegi A. Valente M. T. Riccioni L. Orzali L. Vitale S.. (2022). New-generation sequencing technology in diagnosis of fungal plant pathogens: A dream comes true?. J Fungi (Basel) 8, 737. doi: 10.3390/jof8070737 PMC9320658 35887492

[B3] Baldrian P. Lepinay C. (2021). High-throughput sequencing view on the magnitude of global fungal diversity. Fungal Diversity 114, 539–547. doi: 10.1007/s13225-021-00472-y

[B4] Bandara H. Samaranayake L. P. (2019). Viral, bacterial, and fungal infections of the oral mucosa: Types, incidence, predisposing factors, diagnostic algorithms, and management. Periodontol 80, 148–176. doi: 10.1111/prd.12273 31090135

[B5] Bhattacharya S. Sae-Tia S. Fries B. C. (2020). Candidiasis and mechanisms of antifungal resistance. Antibiot (Basel) 9, 312. doi: 10.3390/antibiotics9060312 PMC7345657 32526921

[B6] Campbell L. J. Walsh D. P. Blehert D. S. Lorch J. M. (2020). Long-term survival of pseudogymnoascus destructans at elevated temperatures. J. Wildl Dis. 56, 278–287. doi: 10.7589/2019-04-106 31622188

[B7] Cannon R. D. (2022). Oral fungal infections: past, present, and future. Front. Oral. Health 3, 838639. doi: 10.3389/froh.2022.838639 PMC8850356 35187534

[B8] Centers for Disease Control and Prevention (CDC) (2014). Revised surveillance case definition for HIV infection–United States 2014. MMWR Recomm Rep. 63, 1–10. 24717910

[B9] Chakladar J. John D. Magesh S. Uzelac M. Li W. T. Dereschuk K.. (2022). The intratumor bacterial and fungal microbiome is characterized by HPV, smoking, and alcohol consumption in head and neck squamous cell carcinoma. Int. J. Mol. Sci. 23, 13250. doi: 10.3390/ijms232113250 36362038 PMC9655846

[B10] Chang S. Guo H. Li J. Ji Y. Jiang H. Ruan L.. (2021). Comparative analysis of salivary mycobiome diversity in human immunodeficiency virus-infected patients. Front. Cell Infect. Microbiol. 11, 781246. doi: 10.3389/fcimb.2021.781246 PMC8671614 34926323

[B11] Chen X. Cao Y. Chen M. Wang H. Du P. Li H.. (2022). HIV-infected patients rarely develop invasive fungal diseases under good immune reconstitution after ART regardless high prevalence of pathogenic filamentous fungi carriage in nasopharynx/oropharynx. Front. Microbiol. 13, 968532. doi: 10.3389/fmicb.2022.968532 PMC9666755 36406455

[B12] Chen X. Zhou X. Liao B. Zhou Y. Cheng L. Ren B. (2021). The cross-kingdom interaction between *Helicobacter pylori* and *Candida albicans* . PloS Pathog. 17, e1009515. doi: 10.1371/journal.ppat.1009515 33956895 PMC8101746

[B13] Chu L. Shu Z. Gu X. Wu Y. Yang J. Deng H. (2023). The endocannabinoid system as a potential therapeutic target for HIV-1-associated neurocognitive disorder. Cannabis Cannabinoid Res. 8, 445–463. doi: 10.1089/can.2022.0267 36745405

[B14] Crater J. M. Nixon D. F. Furler O’Brien R. L. (2022). HIV-1 replication and latency are balanced by mTOR-driven cell metabolism. Front. Cell Infect. Microbiol. 12, 1068436. doi: 10.3389/fcimb.2022.1068436 PMC9712982 36467738

[B15] d’Enfert C. Kaune A. K. Alaban L. R. Chakraborty S. Cole N. Delavy M.. (2021). The impact of the Fungus-Host-Microbiota interplay upon *Candida albicans* infections: current knowledge and new perspectives. FEMS Microbiol. Rev. 45, 1–55. doi: 10.1093/femsre/fuaa060 PMC8100220 33232448

[B16] Devi K. S. R A. Kizhakkoottu S. (2024). Clinico-demographic parameters of oral fungal infections: an institutional retrospective study. Cureus 16, e55386. doi: 10.7759/cureus.55386 38562320 PMC10983061

[B17] Erfaninejad M. Zarei Mahmoudabadi A. Maraghi E. Hashemzadeh M. Fatahinia M. (2022). Epidemiology, prevalence, and associated factors of oral candidiasis in HIV patients from southwest Iran in post-highly active antiretroviral therapy era. Front. Microbiol. 13, 983348. doi: 10.3389/fmicb.2022.983348 PMC9478364 36118210

[B18] Fidel P. L. Jr Thompson Z. A. Lilly E. A. Granada C. Treas K. Dubois K. R. 3rd. (2021). Effect of HIV/HAART and other clinical variables on the oral mycobiome using multivariate analyses. mBio 12, e00294–e00221. doi: 10.1128/mBio.00294-21 33758093 PMC8092233

[B19] Ghannoum M. A. Jurevic R. J. Mukherjee P. K. Cui F. Sikaroodi M. Naqvi A.. (2010). Characterization of the oral fungal microbiome (mycobiome) in healthy individuals. PloS Pathog. 6, e1000713. doi: 10.1371/journal.ppat.1000713 20072605 PMC2795202

[B20] Govender N. P. Grayson W. (2019). Emergomycosis (*Emergomyces africanus*) in advanced HIV disease. Dermatopathol (Basel) 6, 63–69. doi: 10.1159/000495405 PMC6827448 31700845

[B21] Hager C. L. Ghannoum M. A. (2018). The mycobiome in HIV. Curr. Opin. HIV AIDS 13, 69–72. doi: 10.1097/COH.0000000000000432 29028668 PMC5805152

[B22] Hong B. Y. Hoare A. Cardenas A. Dupuy A. K. Choquette L. Salner A. L.. (2020). The salivary mycobiome contains 2 ecologically distinct mycotypes. J. Dent Res. 99, 730–738. doi: 10.1177/0022034520915879 32315566 PMC7243416

[B23] Isidoro-Ayza M. Klein B. S. (2024). Pathogenic strategies of *Pseudogymnoascus destructans* during torpor and arousal of hibernating bats. Science 385, 194–200. doi: 10.1126/science.adn5606 38991070 PMC12289231

[B24] Jaqua E. Labib W. Danji K. (2022). HIV-associated conditions in older adults. Cureus 14, e32661. doi: 10.7759/cureus.32661 36660505 PMC9844266

[B25] Lopes J. P. Lionakis M. S. (2022). Pathogenesis and virulence of *Candida albicans* . Virulence 13, 89–121. doi: 10.1080/21505594.2021.2019950 34964702 PMC9728475

[B26] Lu S. Y. (2021). Oral candidosis: pathophysiology and best practice for diagnosis, classification, and successful management. J. Fungi (Basel) 7, 555. doi: 10.3390/jof7070555 34356934 PMC8306613

[B27] Madani W. Grayson W. (2022). A presumptive case of cutaneous emergomycosis in a female patient with HIV - Maseru, Lesotho. S Afr J. Infect. Dis. 37, 415. doi: 10.4102/sajid.v37i1.415 36338194 PMC9634954

[B28] Metsch L. R. Pereyra M. Messinger S. Jeanty Y. Parish C. Valverde E.. (2015). Effects of a brief case management intervention linking people with HIV to oral health care: project SMILE. Am. J. Public Health 105, 77–84. doi: 10.2105/AJPH.2014.301871 24832421 PMC4265910

[B29] Mukherjee P. K. Chandra J. Retuerto M. Sikaroodi M. Brown R. E. Jurevic R.. (2014). Oral mycobiome analysis of HIV-infected patients: identification of Pichia as an antagonist of opportunistic fungi. PloS Pathog. 10, e1003996. doi: 10.1371/journal.ppat.1003996 24626467 PMC3953492

[B30] Mukherjee P. K. Chandra J. Retuerto M. Tatsuoka C. Ghannoum M. A. McComsey G. A. (2018). Dysbiosis in the oral bacterial and fungal microbiome of HIV-infected subjects is associated with clinical and immunologic variables of HIV infection. PloS One 13, e0200285. doi: 10.1371/journal.pone.0200285 29995962 PMC6040710

[B31] Mukherjee P. K. Wang H. Retuerto M. Zhang H. Burkey B. Ghannoum M. A.. (2017). Bacteriome and mycobiome associations in oral tongue cancer. Oncotarget 8, 97273–97289. doi: 10.18632/oncotarget.21921 29228609 PMC5722561

[B32] Mushi M. F. Bader O. Taverne-Ghadwal L. Bii C. Groß U. Mshana S. E. (2017). Oral candidiasis among African human immunodeficiency virus-infected individuals: 10 years of systematic review and meta-analysis from sub-Saharan Africa. J. Oral. Microbiol. 9, 1317579. doi: 10.1080/20002297.2017.1317579 28748027 PMC5508360

[B33] Mutalik V. S. Bissonnette C. Kalmar J. R. McNamara K. K. (2021). Unique oral presentations of deep fungal infections: A report of four cases. Head Neck Pathol. 15, 682–690. doi: 10.1007/s12105-020-01217-0 32889592 PMC8134600

[B34] Muthu V. Agarwal R. Dhooria S. Sehgal I. S. Prasad K. T. Rudramurthy S. M.. (2023). Mucormycosis in human immunodeficiency virus-infected individuals: A systematic review of case reports. Mycopathologia 188, 755–763. doi: 10.1007/s11046-023-00775-5 37501018

[B35] Ngozi A. E. Ifeyinwa O. J. (2022). Prevalence and antimycotic susceptibility profile of Candida species in the oral cavities of HIV/AIDS patients and pregnant women in Nsukka, Nigeria. Malays J. Microbiol. 18, 271. doi: 10.21161/mjm.211293

[B36] Pérez-Cobas A. E. Gomez-Valero L. Buchrieser C. (2020). Metagenomic approaches in microbial ecology: an update on whole-genome and marker gene sequencing analyses. Microb. Genom 6, 1–22. doi: 10.1099/mgen.0.000409 PMC7641418 32706331

[B37] Roomaney I. A. Holmes H. K. Engel M. M. (2021). Treatment of oral fungal infections using photodynamic therapy: Systematic review and meta-analysis. Clin. Exp. Dent. Res. 7, 354–364. doi: 10.1002/cre2.408 33797857 PMC8204034

[B38] Santosh A. Muddana K. Bakki S. R. (2021). Response to commentary: fungal infections of oral cavity: diagnosis, management, and association with COVID-19. SN Compr. Clin. Med. 3, 2205–2206. doi: 10.1007/s42399-021-01033-9 34541457 PMC8439534

[B39] Scholz K. J. Höhne A. Wittmer A. Häcker G. Hellwig E. Cieplik F.. (2025). Co-culture of Helicobacter pylori with oral microorganisms in human saliva. Clin. Oral. Investig. 29, 79. doi: 10.1007/s00784-025-06160-4 PMC11757641 39849235

[B40] Shi T. Li X. Q. Zheng L. Zhang Y. H. Dai J. J. Shang E. L.. (2021). Sesquiterpenoids from the antarctic fungus pseudogymnoascus sp. HSX2-11. Front. Microbiol., 12, 688202. doi: 10.3389/fmicb.2021.688202 PMC8226235 34177873

[B41] Shirtliff M. E. Peters B. M. Jabra-Rizk M. A. (2009). Cross-kingdom interactions: *Candida albicans* and bacteria. FEMS Microbiol. Lett. 299, 1–8. doi: 10.1111/j.1574-6968.2009.01668.x 19552706 PMC4406406

[B42] Shytaj I. L. Procopio F. A. Tarek M. Carlon-Andres I. Tang H. Y. Goldman A. R.. (2021). Glycolysis downregulation is a hallmark of HIV-1 latency and sensitizes infected cells to oxidative stress. EMBO Mol. Med. 13, e13901. doi: 10.15252/emmm.202013901 34289240 PMC8350904

[B43] Sodré C. S. Rodrigues P. Vieira M. S. Marques Paes da Silva A. Gonçalves L. S. Ribeiro M. G.. (2020). Oral mycobiome identification in atopic dermatitis, leukemia, and HIV patients - a systematic review. J. Oral. Microbiol. 12, 1807179. doi: 10.1080/20002297.2020.1807179 32944157 PMC7482892

[B44] Spalanzani R. N. Mattos K. Marques L. I. Barros P. Pereira P. Paniago A.. (2018). Clinical and laboratorial features of oral candidiasis in HIV-positive patients. Rev. Soc Bras. Med. Trop. 51, 352–356. doi: 10.1590/0037-8682-0241-2017 29972567

[B45] Talapko J. Juzbašić M. Matijević T. Pustijanac E. Bekić S. Kotris I.. (2021). *Candida albicans*-the virulence factors and clinical manifestations of infection. J. Fungi (Basel) 7, 79. doi: 10.3390/jof7020079 33499276 PMC7912069

[B46] Vallianou N. Kounatidis D. Christodoulatos G. S. Panagopoulos F. Karampela I. Dalamaga M. (2021). Mycobiome and cancer: what is the evidence. Cancers (Basel) 13, 3149. doi: 10.3390/cancers13133149 34202433 PMC8269322

[B47] Vila T. Sultan A. S. Montelongo-Jauregui D. Jabra-Rizk M. A. (2020). Oral candidiasis: A disease of opportunity. J. Fungi (Basel) 6, 15. doi: 10.3390/jof6010015 PMC7151112 31963180

[B48] Villanueva P. Vásquez G. Gil-Durán C. Oliva V. Díaz A. Henríquez M.. (2021). Description of the first four species of the genus *pseudogymnoascus* from Antarctica. Front. Microbiol. 12, 713189. doi: 10.3389/fmicb.2021.713189 PMC8640180 34867840

[B49] Wu B. Hussain M. Zhang W. Stadler M. Liu X. Xiang M. (2019). Current insights into fungal species diversity and perspective on naming the environmental DNA sequences of fungi. Mycology 10, 127–140. doi: 10.1080/21501203.2019.1614106 31448147 PMC6691916

[B50] Xie Z. Canalda-Baltrons A. d’Enfert C. Manichanh C. (2023). Shotgun metagenomics reveals interkingdom association between intestinal bacteria and fungi involving competition for nutrients. Microbiome 11, 275. doi: 10.1186/s40168-023-01693-w 38098063 PMC10720197

[B51] Zhang W. Cheng H. Gui Y. Zhan Q. Li S. Qiao W.. (2021). Mannose treatment: A promising novel strategy to suppress inflammation. Front. Immunol. 12, 756920. doi: 10.3389/fimmu.2021.756920 PMC8502929 34646279

